# Keep on operating: how to deal with power cuts

**Published:** 2013

**Authors:** Brian Savage

**Affiliations:** Ophthalmologist, Mvumi Hospital, Dodoma, Tanzania. Able4s@gmail.com

**Figure:**
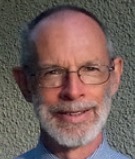
Brian Savage

It is that time of year again: rains have come and gone, it is dry, and it seems the electricity generating industry is coming to another crisis. We are 90 minutes into our main operating list and doing well. Unexpectedly, the music from the radio cuts, and so do the lights from the microscopes. In the sudden silence we realise there is another power cut and the hospital generator is not working. Do we tell the people waiting to come back another day?

## Finishing the present operation

Our first priority is to finish the present operation. A good halogen torch (or other bright, focused light), held by a nurse, may help; however, we have found the following two options even more useful.

1. **Uninterruptible power supply (UPS)**. A UPS (Figure 1), similar to that used for a desktop computer, allows our two microscopes another 30 minutes or so of power so we can conclude the present operation with illumination of better quality than a torch.
2. **Inverter**. The inverter is connected to the mains and charges a lead acid battery connected to it (Figure 2). As soon as the mains power cuts, power from the battery – direct current (DC) – flows through the inverter, and becomes 220v alternating current (AC). It can now power a microscope requiring AC current.

## Continuing an operating list

In a setting where patients have travelled long distances, often at high cost, cancelling an operating list due to a power failure is something to avoid if at all possible. If the hospital generator is not reliable, we strongly recommend that you invest in one of the following.

1. **A small generator**. A small petrol generator, such as the one in Figure 4, should be adequate to run your microscope. If you are buying one specifically for this purpose, it is important to know the total amount of electricity needed by the microscope, vitrector and any other equipment using a small amount electricity, then you can be sure the generator will produce enough.
2. A 12v battery. We have a microscope that has the option of running on 220v AC or 12v DC. A 12v battery (Figure 3) can last for several hours, providing enough power to finish the present operation and to perform additional operations. Alternatives include the battery from the car you arrived in, or a specially-purchased dry cell battery similar to that found in many UPS devices. We have completed many operating lists using this method, but **only** when we have two or more charged batteries available (suitable UPS batteries cost around US $30).

**Figure:**
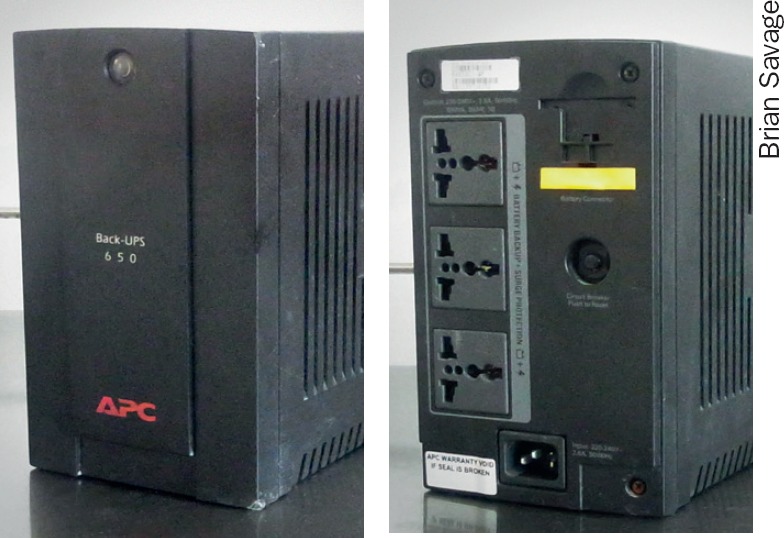
Figure 1. UPS, front and rear views showing power outlets

**Figure:**
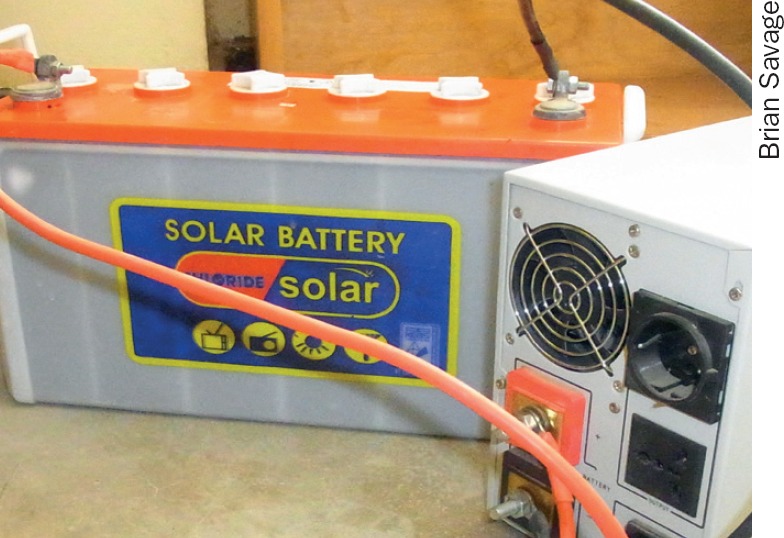
Figure 2. Inverter, attached to lead acid battery (chargeable by solar or mains electricity)

**Figure:**
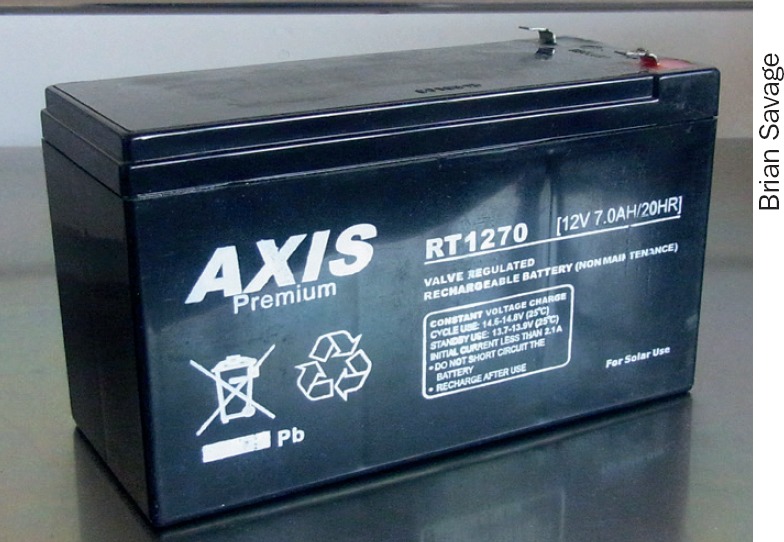
Figure 3. 12v 7amp-hour battery for direct current

**Figure:**
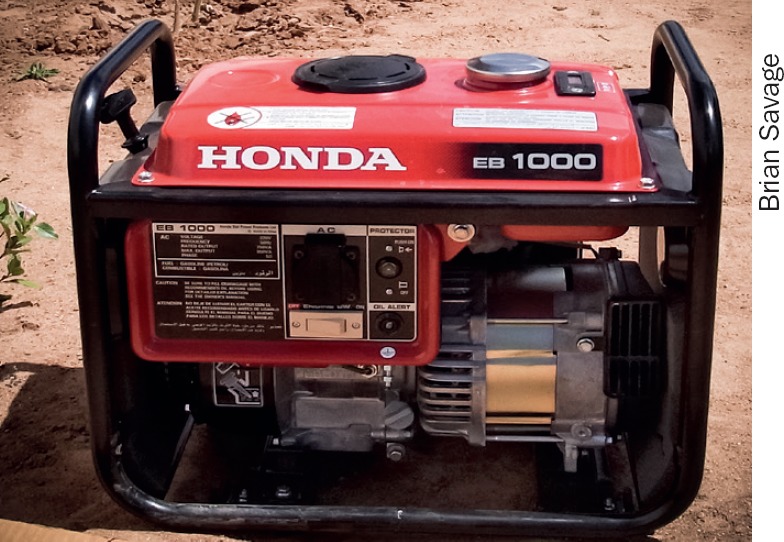
Figure 4. Small petrol generator

We have had no significant problems using our basic Scan Optics microscope with either the generator or the 12v battery for a list lasting several hours.

### Sterilisation

Problem solved? Well, not quite: the nurses have run out of sterile instruments, which they usually sterilise during the list in autoclaves requiring large quantities of electricity. Our batteries and alternators cannot provide this load. We have developed two different approaches to providing sterile instruments during a power cut.

1. Use pre-packed, pre-sterilised instruments, just as one uses pre-sterilised drapes. This requires advance planning and having several operating sets that can be pre-sterilised and double-wrapped in sterile drapes. Provided the packs remain dry, they can be kept for up to 1 week.
2. Use a domestic pressure cooker (costing around US $70) on top of a gas burner that screws into the top of a gas cylinder (Figure 5). The burner gives quick and effective heat and using a pressure cooker is better and quicker than boiling instruments in a pan. The gas burner with cylinder is readily available locally for around US $100.

These suggestions do not work for all situations. If you have a sophisticated microscope, then a standard UPS will not have the power to give a consistent, steady illumination. Also, you may not have sufficient operating instruments to pre-pack and sterilise.

However, if you want your operating to go well through a period of power shortage, and are prepared to plan for this in advance, you might like to give these suggestions a try. It is possible to continue operating during a power cut and the equipment to enable this is usually available locally.

**Figure:**
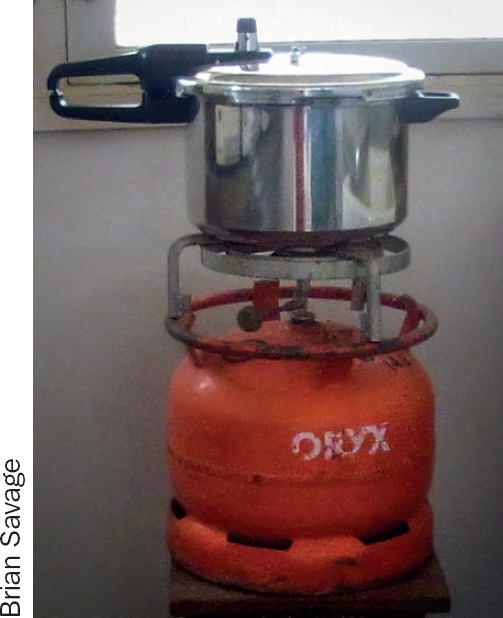
Figure 5. Cylinder-mounted gas burner with domestic pressure cooker

